# "The Hospital" Nursing Section

**Published:** 1906-09-08

**Authors:** 


### The Hospital.

Nursing Section.

Contributions for "The Hospital," should be addressed to the Editor, "The Hospital" Nursing Section, 28 & 29 Southampton Street, Strand, London, W.C.

No. 1,042.—Vol. XL. SATURDAY, SEPTEMBER 8, 1906.

### Notes on News from the Nursing World.

### THE VALUE OF FIRE-DRILL.

On Sunday a fire broke out at Northampton General Hospital, and once more a striking illustration of the value of fire drill was afforded. There is a fire drill every week at Northampton Hospital, and the whole of the staff are carefully instructed as to their duty in the event of a conflagration. When the outburst was discovered on Sunday the sisters and nurses at once connected their hoses and turned on the water supply, while three of the medical staff ascended the ladder to the roof and applied the hoses. The patients in the medical ward were quickly removed to places of safety by the nurses, under the superintendence of the matron, by means of the fire-escape staircase to the ward below. There was no panic and no confusion, and none of the patients were any the worse for the incident. Throughout the trying period everyone worked splendidly, and there is no doubt that the results of the outbreak would have been much more serious if the individual members of the staff had not promptly taken up their positions immediately the alarm was given. Several of the patients who were removed from the medical ward, being in a fit state, were sent home, and the others were transferred to the emergency ward, where they will remain until the necessary repairs are completed. We warmly congratulate Miss Densham and her staff on the help they rendered on the occasion and the presence of mind which they displayed from the moment it was intimated that fire had broken out.

### GERMAN NURSES IN ENGLAND.

A fortnight ago we published an article by a contributor who alleged that there is a scarcity of nurses in Germany, and gave her reasons for the difficulty experienced by German institutions in obtaining them. She also, as we pointed out, accounted for the increasing number of German nurses in England. The lay press now affirm that there is an "invasion of alien nurses" in London. If, as we suspect, this statement is only founded upon our contributor's article and our accompanying remarks, the language of exaggeration or elaboration has been resorted to. So far as we are aware, there is no "invasion" of alien nurses either in London or in any part of the United Kingdom. The number of German nurses in the country is larger than it was, but most of these have been trained here, and work entirely on English methods.

### THE CARE OF PAUPER IMBECILES.

It is obviously wrong that an imbecile woman, certified as "not safe," should be in charge of two inmates, themselves both weak-minded, and the engagement, at the instance of the medical officer of the Auckland Workhouse, of two trained nurses to attend to the three women may, at first sight, appear extravagant. If, as it seems clear, these patients require constant supervision, their proper permanent place is not the wards of the Workhouse. We always favour the employment of an adequate staff of trained nurses in attendance upon the sick poor, but in this instance the patients, if really dangerous, should be removed as soon as possible to the county asylum, where they would be suitably nursed at much less cost to the ratepayers.

### THE COTTAGE HOSPITAL MATRON.

The difficulties of working a cottage hospital on up-to-date lines are pointed out in our columns to-day by a matron whom, we happen to know, has herself been successful. We entirely agree with her that the larger institutions of this character should not be contemplated unless there are means sufficient to pay a decent salary for a fully trained matron, to give her a fully trained assistant nurse, and to provide the wages of two good servants. A trained nurse who undertakes the position of matron at a salary of £25 a year with one servant to assist her is not likely to do justice to either herself or her patients, and we are sure that the chances of it leading to better things are more slender than the chances that it will ruin her career. On the other hand, the matron of a cottage hospital, with adequate help, able to carry on her work in a proper manner, can scarcely fail to advance, for the experience she gains constitutes an excellent qualification for a higher office.

### THE IGNORANT MIDWIFE STILL ABROAD.

The medical officer of health for the South Molton Union District has issued a report which shows that the Midwives Act is misunderstood, or ignored, in some of the rural districts. He states that the Burrington parish nurse is the only one registered in the Union who has had any special training, and who is sufficiently acquainted with the duties of a midwife to discharge them in a proper manner. As to the other midwives he does not complain concerning the condition of their houses and general cleanliness, but he had to tell a few of them that if they wish to continue to act as midwives they must be more careful. The most serious part of his report is "that the large majority of them are more or less unaware of the requirements of the Central Midwives Board." He adds that, "with one or two exceptions, they informed him that they did not understand the use of the instruments," and that "when he explained the necessity for keeping a proper register of the cases he found that they had not the necessary knowledge to answer the questions." He thinks that to strictly enforce the regulations in these cases, to require the midwives to use disinfectants or instruments they do not understand would be like playing with fire. Later on he proposes to pay another visit to the midwives in order to ascertain how far the instructions he has given them have been carried out. Meanwhile, the District Council have sent a copy of the report to the County Council who are charged with the administration of the Act in Devonshire; and this is all that they can do. But if the Devonshire County Council do not take action the Midwives Board must intervene.

### THE OBLIGATIONS OF GUARDIANS.

It is not usual for the parents of a probationer to ask that her engagement may be terminated before the period for which she has agreed to serve is completed. But the father of a young woman who is being trained at Chichester Poor-law Infirmary had exceptional reasons for requesting that his daughter might be permitted to leave at the end of six months. He affirmed that no lectures were given by the medical officer, and that his daughter was chiefly employed to look after lunatics, one of whom had kicked and bitten her. It is admitted that, though the Guardians promise that lectures shall be given by the medical officer, they do not pay him for lecturing, and he cannot therefore be blamed. But unless Guardians fulfil their obligations they must expect to experience difficulties with their probationers. The inquiry which it has been decided to hold at Chichester by the Visiting Committee seems a day behind the fair.

### THE RESIGNATIONS AT AN IRISH INFIRMARY.

The resignation of the superintendent nurse and two assistant nurses of their positions at the Workhouse Infirmary under the control of the Banbridge Guardians is this month to form the subject of an inquiry by an inspector of the Irish Local Government Board. The three nurses, it will be recollected, declared their readiness to withdraw their resignations if another nurse in the Infirmary would accept three weeks' leave of absence while an official investigation was taking place. But as she declined to do so they adhered to their decision, and have since quitted the building. Many rumours are current as to the cause of the friction, and it is very important that the facts of the case should be authoritatively ascertained.

### STEPNEY AND MALTA.

We suggest to the authorities of Malta, who are extremely alarmed at the extraordinary mortality among infants—the percentage of deaths of children under a year old being at the rate of 244 for every 1,000 born in the year—that trained nurses should be appointed as inspectors of health. In England the experiment has been made with signal success. For example, in the borough of Stepney, where a female health inspector was engaged a couple of years ago because of the high rate of infantile mortality, there has been a marked improvement, and it has just been determined to retain the services of Miss Forrester for a further term of two years. As the report of the medical officer for Stepney states, the reduction of the number of deaths in the Limehouse district from 185 per 1,000 births to 102, as the result of continuous teaching by the health visitor in the matter of feeding of infants, is a convincing practical proof of her value. If, as is probably the case, a great number of the deaths of infants in Malta is due to ignorance, the engagement of qualified women as health visitors might speedily effect an alteration.

### A HINT TO SCHOOL NURSES.

The offer of the Birmingham City Dental Hospital to undertake the inspection of the teeth of scholars attending some of the public elementary schools and to do certain necessary work for the preservation of the teeth, suggests a hint on this subject to school nurses. The school nurse can do much with the children; she can impress upon them in her rounds of inspection that cleanliness of the mouth is an essential part of personal cleanliness, and that decayed teeth and fermenting fragments of food are a potent factor in the production of numerous gastric and intestinal troubles. It is true that we occasionally meet with specimens of hale old men whose teeth can crack a nut with the best, and who have never handled a toothbrush in their lives; but these are exceptions. Their great protection lies in the hard food they eat—dry crusts and green apples to wit—which perform the same service for them as bones for dogs and other carnivorous animals, scraping and cleaning the teeth. It would be an advantage if small prizes could be offered—say twice a year—to those children who achieved a standard record of personal cleanliness in the matter of teeth, hair, or nails. Without necessarily tending to cultivate vanity, it would give a certain impetus to the feeling of proper pride in personal appearance and lead to a better appreciation of neatness and order than obtains among the youth of the present day working classes.

### THE DISTRICT NURSE IN THE ISLE OF WIGHT.

District nursing is making excellent progress in the Isle of Wight. Of course, it is not, in any sense, a novelty. For example, seventeen years have elapsed since the Newport District Nursing Society was formed, and at the last meeting which has just been held, it transpired that the number of cases attended by the nurses during the twelve months was larger than on any previous occasion. As showing how the press can assist, we note that it is stated in the report that there would have been a deficiency if the *Isle of Wight County Press* had not drawn attention to the needs of the organisation, with the result that a few kind friends contributed liberally. The balance on the right side was, therefore, upwards of £10. Even so the receipts were £5 less than in the previous year, and the expenditure £22 more. But there is no reason to suppose that there will be any serious difficulty in maintaining the existing staff. In fact, there is no more popular institution in the island than the district nurse, and the appreciation of her work has materially increased since maternity cases have become an important feature.

### A COMPLIMENT TO MISS SIDNEY BROWNE.

WE are pleased to learn that Miss Sidney Browne, R.R.C., has been granted permission to retain the badge of Queen Alexandra's Imperial Military Nursing Service, "in recognition of her long and meritorious service." The late matron-in-chief thoroughly deserves the compliment which has been paid to her, but it will not diminish the regret which is felt that her association with the Service ceased at a time when her abilities and her energy are absolutely unimpaired.

### QUEEN ALEXANDRA'S MILITARY NURSING SERVICE.

WE understand that Miss C. D. E. F. Dunn has resigned her appointment as staff nurse in Queen Alexandra's Imperial Military Nursing Service, and that Miss M. A. Cachemaille, Miss M. E. Medforth, and Miss F. J. Mitchell have been appointed staff nurses provisionally.

### PROGRESS OF THE INDIAN NURSING ASSOCIATION.

IT is gratifying to learn that the efforts of Lady Minto to place the Indian Nursing Association on a satisfactory financial basis are exciting general interest. Small donations are coming in well from the poorer classes, including some from provinces which are not for the present included within the scope of the operations of the organisation. The Punjab Trades Association are taking up the scheme warmly, and a circular letter from the President has been issued asking for the support of members. At Quetta there has been a meeting under the auspices of the Governor-General, at which a resolution was passed in favour of the selection of Quetta as a nursing centre for Baluchistan, with at least two permanent and liberally paid trained nurses. The fund, which in July amounted to about 25,000 rupees, is to be kept open until the end of December.

### GLASGOW LOCK HOSPITAL.

AN enjoyable entertainment was given at the Glasgow Lock Hospital last week by the staff and patients, consisting of songs, tableaux, and a sketch. The occasion was the return of the matron from her holiday, who was presented with a handsome gold bracelet as a token of appreciation for all she has done for the benefit of the hospital and the uplift of the patients during her three years' superintendence.

### ENTERTAINMENT TO SUSSEX NURSES.

ON Thursday last week the nurses affiliated to the Sussex County Nursing Association working in West Sussex were invited by the Countess of March to spend the afternoon at Molecombe. Seven nurses, with Miss Attenborough, inspector of midwives, West Sussex; Mrs. Barford, Selsey; Miss Holbrook and Miss Dyer county superintendents, Lincoln and Sussex, were able to be present. Lady Gifford, of Old Park, Chichester, met the party at the station, and drove them to Goodwood, where they were received by Lady March. They proceeded to Molecombe for tea, and spent some time in the grounds. An enjoyable afternoon was brought to a close by the drive back to Chichester Station.

### EXAMINATION AT LAMBETH INFIRMARY.

AT the last meeting of the Lambeth Guardians the Chairman presented the probationers at the Infirmary who lately passed their examination with their certificates. Out of twenty-four candidates nineteen were successful. Miss King obtained the first place and the prize, and the other nurses who passed were the Misses Davies, Chaloner, Cox, Horner, Sunter, Skinner, Dexter, Phillips, Chard, C. Walker, M. Walker, Gibbs, Keaveny, Pridmore, Seigel, Buller, Bayne, and McDonald. Mr. F. C. Abbott, of St. Thomas's Hospital, who conducted the examination, stated that the candidates as a whole showed themselves well taught, and the Chairman congratulated the matron and the staff upon the result.

### SALARIES IN TASMANIA.

ACCORDING to statistics supplied by the Secretary of the Hobart General Hospital at a recent meeting of the Board, the probationers in most of the Tasmanian hospitals received up to 1903 a salary of £15 the first year, £20 the second, and £25 the third. In 1903 the maximum salary has been £30, but this is due to the addition of a fourth year of training. The salaries of the sisters commence at £40, and are increased at the rate of £5 a year up to £55, the senior sister receiving £60. The Secretary states that these salaries compare favourably with, and are slightly higher than, those paid in the public hospitals of the other Australasian States.

### BEQUESTS TO NURSES.

UNDER the will of the late Mr. W. L. Saunder, for many years general superintendent and secretary of the Manchester Royal Infirmary, Miss Florence M. Calvert, superintendent of nurses at the Infirmary, and Miss Ellen Stephens, one of the sisters, receive £500 each.

### SHORT ITEMS.

THE name of the nurse attached to the Borough Hospital, Southport, who recently saved the life of a bather at Douglas, Isle of Man, is Wrigley, not Wright.—On Saturday last the Duchess of Northumberland entertained about a hundred and fifty members of the Northumberland County Nursing Association at Alnwick Castle, including some 70 nurses.—The substantial sum of £25 has been realised by a garden fête and fancy dress dance last week in aid of the Holbeach District Nursing Association.—It having become necessary to find larger premises for the accommodation of pupils, the Maternity Nursing Association has removed from 5 Little James Street, W.C., to 63 Myddelton Square, Clerkenwell, E.C.

### The Nursing Outlook.

"From magnanimity, all fears above;
From nobler recompense, above applause,
Which owes to man's short outlook all its charm."

### THE EVILS OF STATE INTERFERENCE.

The *Cottage Nurse* in its current issue publishes a vigorous protest by the honorary secretary against "reckless, feverish haste to amend all evils by act of Parliament." Such a course, such a policy "is the creation of a host of bureaucrats empowered through boards, county councils, and offices to carry out State interference. Why cannot people be content to work on the good old English lines of helping their neighbours without interfering with them too much?" Our forbears and sires, who, it must be remembered, did much if not all the foundation building of the British Empire, held, as we think wisely, that free men and women should keep themselves free from State interference of every possible kind. The honorary secretary of The Cottage Benefit Nursing Association has, therefore, historical warrant for her screed.

The crux of the matter appears to be this. The Cottage Benefit Nursing Association trains and supplies cottage nurses for country districts, unites all nursing associations working on the Holt-Ockley system for mutual assistance and combined action, keeps a register of cottage nurses, superintends cottage nurses on the register in their duties, and encourages and helps them in sickness and difficulties. Miss Broadwood maintains that her Association has proved the possibility of working upon these non-pauperising lines, and that in future the Association will have to fight for its rights to do even this. State interference is being directed against the Cottage Benefit Nursing Association "by persons jealous of its success," and "ambitious for professional and official recognition and distinction." Miss Broadwood, therefore, urges the members of her Association "to unite at once and show their strength, to support the central organisation, and through its branches to give timely counsel and substantial help where more vigorous management is essential, to maintain the standard level of training for cottage nurses, to encourage good women to become cottage nurses by safeguarding them from overwork, professional contempt and State interference, and to perfect a sympathetic, practical, legal, just, and automatic control of cottage nurses." Miss Broadwood is seriously alarmed by the dangers which threaten the work of the Cottage Benefit Nursing Association. She declares "these dangers are so real that no language can exaggerate them." Hence she issues her fighting address and declares "my fighting life, if measured by the psalmist's span, is but ten years more; but by our constitution, and by the fact that we have amongst our working secretaries others as energetic as myself, no one need fear that there will ever lack good fighters amongst us who will not fear to face man clothed in a little brief authority." It would be well, we think, for the higher interests of nurse training in this country if a little more of the fighting spirit could be instilled into those responsible for the management of the nurse-training schools.

Miss Broadwood expresses supreme contempt for State registration in every form. "It was the ambitious lady midwives, who had been agitating for the last twenty-two years to exalt their profession, that forced through the not too well-considered Midwives Act, and now it is a clique of ex-nurses who are very near indeed to forcing State registration of nurses upon the country." "The dire necessity of all big hospitals will almost certainly result in their becoming nationalised, and then nurses trained and employed in them will become civil servants under the control of bureaucrats." Miss Broadwood takes it as a matter of course that "the hospitals will become Government training schools, and then woe betide the training homes of the Cottage Benefit Nursing Association, for a Government Board will have the power to determine what is or is not a school, and the cast-iron pattern of Government nurses will be abroad. Will this Government nurse minister comfort and relief to the poor who will be always with us?"

Miss Broadwood defines State registration as "some board, some council, or some office with powers to make rules and to enforce them with an independence of control denied to individual citizens. England has yet to gauge the full amount of tyrannical folly that can be perpetrated under this bureaucratic system. It will probably take a generation or two of suffering to quench the present mania for State interference, to muzzle the demon of State control, and the growth of bureaucratic tyranny. Unfortunately at the moment the shackles of State interference are being forged very fast indeed by those who are aided and abetted in every possible quarter by the evil spirits of self-interest, professionalism, and ambition." Seeing that we have all felt enervated by the heat of late it should do us good to read so stirring a protest against paternal government in all its forms.

### Abdominal Surgery.

By Harold Burrows, M.B., F.R.C.S., Assistant Surgeon to the Seamen's Hospital, Greenwich, and to the Bolingbroke Hospital, Wandsworth Common.

### TUBAL PREGNANCY.

Normal pregnancy commences with the passage of an ovum from the ovary along the Fallopian tube to the uterus. After fertilisation, the ovum becomes attached to the wall of the uterus and begins to develop.

But it happens occasionally that the ovum is arrested in the Fallopian tube, and, becoming fertilised, commences to grow in this situation. This mishap is described as tubal pregnancy, extra-uterine gestation, or ectopic gestation.

The condition is dangerous, because the Fallopian tube is not able to expand sufficiently to contain the growing embryo, and consequently in the course of time the Fallopian tube bursts and hæmorrhage ensues. The tube may rupture directly into the peritoneal cavity or else into the broad ligament.

In the former case the hæmorrhage is apt to be very severe, but if the rupture is into the broad ligament the bleeding is less copious, being checked by the pressure of the surrounding tissues.

In either case, after rupture of the tube the embryo may die. When this happens the effused blood may become absorbed and the patient may recover completely. In other instances the effused blood becomes infected, and gives rise to dangerous inflammation and suppuration in the pelvis. Or the blood becomes absorbed without suppuration taking place, and yet the patient remains more or less crippled by the presence of adhesions which interfere with the functions of the pelvic organs.

If the embryo does not die, and the patient is not relieved by an operation, further trouble is certain to arise. Either there will be fresh attacks of hæmorrhage until the fœtus or the parent succumbs, or else the former will continue to grow and perhaps will reach its full term of development lying free in the peritoneal cavity—the Fallopian tube in which it lay at first having ruptured in the early stage of pregnancy as already mentioned. It is manifest, in this case, that the child lying free in the abdominal cavity instead of in the uterus, cannot be born in the natural manner, and indeed cannot be born at all unless the abdomen is opened for its removal—an operation that has a high mortality, chiefly on account of the difficulty of removing the abnormally situated placenta. This operation is not to be confused with Cæsarean section which is performed for removal of a child from the uterus when the child cannot be born naturally owing to the narrowness of the pelvic outlet.

It will be seen that ectopic pregnancy is a condition set about with perils for the unfortunate mother; but there is this consolation that a skilful and well-timed operation is nearly sure to bring the patient safely through her trials. For the cases are comparatively rare in which the primary loss of blood is so severe as to be fatal before there has been time to operate upon the patient and stop the bleeding. It is remarkable how patients, even though they appear to be nearly *in extremis* from hæmorrhage, will recover from the operation.

With regard to the symptoms, there is seldom anything to suggest the presence of ectopic pregnancy until rupture of the tube ensues. The patient may or may not at the time believe herself to be pregnant, but rupture often takes place before sufficient time has elapsed for her to be sure about the matter.

At the moment when rupture occurs the patient experiences a sudden acute pain in the abdomen and soon becomes faint. The abdomen becomes tender and rigid, especially in the lower part, and the case may resemble one of acute peritonitis. A discharge of blood from the vagina is present usually and is a help towards making a correct diagnosis, although it may lead to an erroneous impression that the case is one of abortion.

In any case where a suspicion of hæmorrhage due to ectopic pregnancy arises the patient should be kept absolutely quiet in bed until the doctor arrives. On no account must brandy or other stimulant be given with the idea of relieving the patient's faintness—such stimulants are more likely to encourage the continuance of hæmorrhage than to arrest it, and hæmorrhage is the paramount danger in these cases.

Immediate laparotomy will probably be required, provided that circumstances are favourable for a surgical operation of this kind. The main objects of such an operation are three—namely, to check the hæmorrhage, to remove the effused blood, and to remove the affected tube and its contents so that no subsequent trouble will arise.

If an immediate operation is impossible for any reason, it will be necessary to keep a careful watch for the appearance of any complications, such as pelvic inflammation, renewed hæmorrhage, and continuance of the signs of pregnancy. At the same time the patient ought to be removed into circumstances where the benefits of surgery will be available if required.

### Cysts and Tumours of the Ovary.

Non-malignant growths of the ovary are comparatively common. They may cause disturbance of menstruation—menorrhagia or amenorrhœa—they frequently produce pain, especially at the menstrual periods, and as they increase in size they are apt to interfere with the functions of the rectum and bladder. In the course of time they grow up out of the pelvis into the abdomen, and it happens not infrequently that the patient first seeks advice because she has accidentally discovered a tumour in the abdomen, or because the abdomen is increasing in size.

It is necessary to remove ovarian tumours by operation in nearly every case as soon as their presence is recognised, because if allowed to remain they will continue to grow, and also because they are liable to certain complications, including simple inflammation with formation of adhesions, suppuration, rupture, and torsion.

These complications may cause sudden and severe abdominal pain and alarming symptoms, not very different from those accompanying other acute abdominal disorders.

### Ruptured Uterus.

Rupture of the uterus is an occasional complication of child-birth, and occurs especially in cases where labour is difficult and prolonged.

The accident is accompanied by severe pain and shock. The placenta, and even the child, may pass through the rent in the uterus into the peritoneal cavity, and coils of intestine may prolapse through the rent into the vagina.

If the child and the placenta are born, and the rent is a small one and not occupied by any portion of the abdominal organs, it may sometimes be treated by plugging with gauze from the vagina. But in other cases it is necessary to open the abdomen, and the uterus having been emptied to stitch up the rent in the uterine wall.

There is a great deal of shock in these cases, and a thorough knowledge of how to treat shock is absolutely necessary for the proper nursing of this as of all other cases which fall within the scope of abdominal surgery.

### The Nurses' Clinic.

### THE DISTRICT NURSE AND DIPHTHERIA, CHICKEN-POX, AND MUMPS.

Diphtheria is found at all seasons, but most commonly in winter and spring. It was formerly regarded as a rural disease, but is now most distinctly an urban one. It is said to prefer low-lying, insufficiently drained places, and towns near a river which frequently overflows. Some families are so curiously susceptible to it that the members will probably be attacked, even if they are living many hundreds of miles apart and under widely varying conditions. Diphtheria occasionally appears in conjunction with scarlet fever and measles.

Poverty and the discomforts attending it are specially favourable to the disease, but it sometimes attacks the strong, healthy, and well-to-do. It affects persons of both sexes and all ages; but although it is not possible at present to say at what age it is most frequently contracted, it is, nevertheless, decidedly a disease of infancy and early childhood. It has been known, however, to spread from a child of two months old to its nurse, parents, and grandparents.

Diphtheria is highly contagious, most probably through the sputum. If the larynx and trachea are affected the patient is in great danger; if the disease reaches the bronchi he invariably dies.

The majority of fatal cases occur among patients under five years of age, while in children under two it is almost inevitably fatal. For this reason the same warnings must be given to mothers as in relation to scarlet fever.

Pneumonia is occasionally found, and there is sometimes a slight amount of bleeding in lungs, heart, and kidneys. Paralysis sometimes, though rarely, follows diphtheria, generally showing itself in the first month. The part most likely to be affected is the soft palate. The signs are : the patient speaks in an altered voice and has difficulty in swallowing. The paralysis may affect (in turn) almost any part of the body, but although it causes prolonged inconvenience it generally ends in recovery, and no special treatment is required.

Antitoxin has greatly reduced the severity of the illness and its consequent mortality, but its effect is considerably lessened if not applied early, and unfortunately in district work the disease is often fully developed before outside assistance is sent for. In the opinion of the poor, pain and disease are strictly proportioned to one another, and in the early stages of diphtheria there is often little pain or even inconvenience, and the onset is extremely gradual. The characteristic symptom is the diphtheritic throat : the uvula, fauces, edges of the soft palate, the tonsils, and the pharynx will be found much reddened and congested, and there is a sprinkling of greyish white specks, which can easily be removed. The specks join into large patches, and what is called a false membrane is formed, and sometimes spreads as far as the trachea or even the bronchial tubes. Ulceration, the formation of pus, or a foul discharge from the nostrils point to malignant scarlet fever, not diphtheria. The two diseases so often occur in conjunction that there is likely to be a slight confusion as to which symptoms are the truly characteristic ones. If a false membrane has formed, no disease but diphtheria can have caused it. The membrane, as a rule, only forms on the parts of the body mentioned above, but may occur on any portion of the body that is bruised, wounded, or blistered.

Cold, damp weather renders recovery more difficult, and fresh, warm, pure air is one of the patient's first needs. The nurse must make a great point of attending to the temperature and ventilation of the room, as the special need for air in these cases is by no means generally understood. The bedding must be light and warm, and hot bottles can be used, but should not be in actual contact with the patient.

The usual fever diet is given, but there is often intense loathing of food. If the patient will not yield to persuasion, or to frequent solicitation to swallow small quantities, nutrient enemata should be given. Force must never be employed, even with very young children, as vomiting and indigestion will probably follow, in addition to the waste of strength caused by angry resistance to such mistaken treatment. All food is given in a liquid form, and consists chiefly of nourishing soups. Stimulants are generally required.

No child should return to school for at least three months after an attack of diphtheria, and should gargle his throat three times daily with an antiseptic throughout this period.

It is not certain that bad drainage can cause diphtheria, but it undoubtedly predisposes people to an attack. The period of incubation varies greatly, as in scarlet fever, and the germs have the same prolonged vitality, and may exist for months in a dormant state. The temperature of the patient is generally between 100 and 102, but may even sink below normal.

*Chicken-pox.*—With ordinary care chicken-pox is not a serious matter, but the patient should be confined to bed, and if the temperature rises much complications should be suspected, although unlikely to occur in any but delicate children. To relieve the irritation of the eruption, boracic ointment may be freely used. One attack confers immunity.

*Mumps*, although not a dangerous disease and free from probable complications, is very painful, and likely to leave the patient in a weak and ailing state, demanding nourishing food and change of air; great care should therefore be taken not to expose children to any risk of infection. Mumps occurs at all times of the year, and although most common among children and very young persons, is often contracted by people of mature age. The same general treatment is required as in other fevers. Incubation varies from seven to twenty-one days, and the illness lasts from seven to fourteen days.

### Incidents in a Nurse's Life.

MY FIRST "NERVE" CASE.

"PLEASE send a quiet, amiable nurse!"

Such was the message received at the Nursing Home to which I belong. I was quite fresh to private nursing and just in from my first case, which had been very successful. It was my turn to go out, and, being considered "quiet and amiable," matron sent me without any misgivings. The patient was a lady suffering from "nerves," which she had had for years, so I fully realised that it would not be all roses unless I made it so myself. On my arrival I was most favourably impressed by the beauty of the house and its surroundings, which was to be my abode for some time to come. The retiring nurse—who looked fagged out—received me. After giving me all instructions she added, "You will not get much rest, nurse; she is always restless. I hope you are a light sleeper, because you are sure to be disturbed several times during the night." "Oh, that does not matter," I said, assuming a cheerfulness I was far from feeling. "I do not mind." Nurse smiled. "Oh, yes, you will, after seven months' broken rest like I have had." "Seven months," I ejaculated, my heart slowly sinking into my shoes. I thanked nurse for her kindness, and bade her good-bye, mentally wondering how long it would be before I were going too.

Up to this time I had not seen my patient, and after my predecessor had taken her departure was left to my own devices for some length of time.

Just as I was contemplating an escape through the window, which looked out on a courtyard some 40 feet below, the door opened and a lady entered, who introduced herself as my patient's companion. After mentioning several details with which I was already acquainted, she informed me that I had better not unpack my luggage, as I might be sent back to the Home again, owing to my future patient's fastidious taste. I was silently wondering what would happen next, when an electric bell rang violently. Off flew the companion in a terrible hurry. Presently she returned. "Mrs. —— will see you now, nurse. I will not come in with you, as she cannot breathe if there are too many people in the room. Don't talk, for she does not like strange voices, and be very quiet, as she cannot bear a noise." I crept into the room like a thief, hardly daring to breathe lest I should be the cause of a fresh attack of "nerves." I was surprised to see a young-looking and not unpleasant-faced lady of about forty years of age, having conjured in my mind's eye a dreadful witch. "Good evening, nurse," she said quite pleasantly, "won't you sit down?" I answered courteously, and drew up a chair where I could see her. Alas! I had done wrong. "Don't sit there," she said crossly, "sit behind the bed." I did so meekly, feeling very much inclined to laugh, when I caught sight of a mirror so placed that she could see anyone in the room. I was saved! I sat down without speaking, and for nearly half an hour "silence reigned supreme." I was just making up my mind to go to sleep, when a voice from the bed called, "You can go now, nurse," adding graciously, "I like you, you are so quiet." I made my escape gladly, wondering if every day would bring a repetition of that dreadful ordeal. The companion was fluttering about in a state bordering on frenzy, and almost embraced me when she saw me leave the room. "What a long time you have been, and what does Mrs. —— say?" she asked breathlessly. "Oh, nothing," I answered laughingly, "except that she likes me because I was quiet." "Oh, I am thankful," she cried; "you don't know what a worry it has been to me." "You poor thing," I thought sympathetically. "I'm sure it must have been," I said, "but I hope it will be all right now." We talked in subdued whispers for some time, and then I went down to dinner. After I had finished she came to me and said, "Mrs. —— will have her dinner now." That passed off successfully, and on removing the tray, to save the housemaid, I thought I would carry it downstairs, but, unhappily, instead of doing so I fell down myself. Oh, the clatter! plates in one direction, knives and forks in another, until a thought the whole household would arrive to see what was the matter. As I sat among the debris in an uncontrollable fit of laughter, the bell rang, and away I had to fly. To my intense surprise my patient had not heard the noise, and only required me to darken the room. I was then told I could go until she rang again. I returned to her an hour later, and after a toilet lasting two hours, I slipped off to bed, more tired than I had ever been in hospital. I had not been in bed half an hour when "ding" went the bell, and I had to stay with her nearly two hours, rubbing her to sleep. Again I slipped off, only to be called after an hour to give her a drink, and two hours later to light the fire, when it was too late to return to bed.

I stayed with my patient for seven weeks, and we became great friends. I talked to her, told her stories of life abroad, of life in hospital, sang to her, read to her, and, on the whole, had quite a happy time. But that bell! I can hear it now, for she rang night after night, until in desperation I had to ask to be recalled. We had an affectionate parting, and she told me that if ever she was "ill" she would have me to nurse her, as I was "the brightest and quietest nurse she had ever had."

### A French War with Consumption.

BY A CORRESPONDENT.

MLLE. CHAPTAL is devoting her life to philanthropic works in Paris, and she has especially turned her attention to alleviating the sufferings of the sick poor, more particularly in the direction of combating consumption. She claims that during the five or six years in which she has worked in this direction, she has lessened the number of cases of tuberculosis in the area of her sphere of action by more than one-third. Whereas when she first began her efforts there were about 80 cases of consumption to every 10,000 of the population, the average now is 49 in 10,000. The result is well worth attaining, and the success has been achieved by preventive measures, by the better feeding and housing of the poor, and by educating them in the matter of fresh air and the danger to others of infection from those suffering from the disease. Some of Mlle. Chaptal's schemes in this direction would not quite approve themselves to workers on charity organisation principles, but it must be remembered that the French people have, to a great extent, already acquired the difficult art of thrift through the hard necessities of their case. In a highly protected country like France all provisions are dear, and consequently it is even more difficult for the poor to provide extra nourishment for their sick than it is for our people.

Without knowing the full conditions under which they live, it is perhaps not quite fair to judge whether in the time of illness they do all that they ought to do, or can be expected to do, for themselves. But this question hardly affects Mlle. Chaptal's preventive measures. Her plans of action are simple, but that they are effectual the results show. In the first place Mlle. Chaptal has established a dispensary solely for tuberculosis; this is opened between 5 and 7 o'clock in the evening, so that those who are well enough to be at work can receive medical advice and attention out of work hours. This dispensary is a charity, pure and simple, but of course it might equally be worked on the lines of a provident dispensary. Then in cases where sufficient nourishing food cannot be supplied by the family themselves, Mlle. Chaptal provides it for the patient from the dispensary. Of course the benefit of fresh air is insisted upon, and here comes in, too, the question of prevention. The advantage is inculcated of a separate room for the invalid, and if the circumstances of the family are such that they cannot be expected to provide this for themselves, Mlle. Chaptal has often herself found the wherewithal, and thus tremendously lessened the danger to the patient's relatives. This is a matter which might well be taken up by charitable persons. A house which is sufficiently large for a family under ordinary conditions may yet not be big enough to allow any member of that family to have a room to himself, and if he is suffering from a disease such as consumption, he forthwith becomes a peril to the other occupiers of his bedroom. Then cottage rooms are usually small, and plenty of fresh air is a necessity for a consumptive. Of course treatment in an open air hospital is often beneficial, and removes the source of danger for a time, but it is usually very costly, and it is not every philanthropically-minded individual who can find the means to send a consumptive, in whom he may be interested, to such a place for two or three months, and even then when the invalid comes home, he returns to the same unwholesome conditions of life as before, and all the good of the change is frequently undone in a week or two.

Again, he may not receive all the benefit that was hoped for from the change, and may be sent home in a dying condition, when he is in a state which is the greatest source of danger to those around him. By this time the funds of his charitable friends are probably exhausted, or at any rate it does not occur to them that when the primary object of a cure has failed, there is a still greater field of usefulness in the prevention of the spread of the disease to others.

It would often be far better to pay 1s. or 1s. 6d. a week for a separate room for the invalid where he can be looked after by his own family without being a peril to their health, and where he can carry out the open-air principle for himself. There is another danger which has been met by Mlle. Chaptal's forethought, and that is the risk of contamination in the washing of soiled clothing. Following the example of Dr. Calmette, Directeur of the Institut Pasteur in Lille, Mlle. Chaptal has established on his principles a laundry for the washing of her consumptive patients' clothes. They are charged a small sum—something like 1d. or 2d. a week—and all their linen is washed in this special place, so that there is no danger of contagion to the rest of the family in this respect.

These are not heroic measures; none of them, except perhaps the laundry, entail a vast expenditure of money, and if worked on a careful business basis, they might be largely supported by the recipients of the benefits themselves. It is not claimed by Mlle. Chaptal that she has found a cure for consumption; far from it. Unless people can be persuaded to put themselves under treatment in the very early stages of the disease, the "cure" becomes costly, and often impossible; a lengthy sojourn by the seaside or in the country may prolong life for a few months, but with a return to the stuffy air of an insanitary home, all the good of the change will speedily be undone. Consumption is a disease which thrives in impure surroundings; it is comparatively seldom found in the clean, well-aired and well-nourished homes of the rich. Therefore its cure, and more especially its prevention, points in the direction of good sanitary and atmospheric conditions, and plenty of wholesome food. Much might be done by the giving of good advice in this respect by district nurses and district visitors. The advantages of fresh air in the home should be preached in season and out of season. Many of the poor are beginning to grasp the idea, and open bedroom windows at night are much more common now, especially among the servant class, than they were twenty years ago. Still much remains to be done in this direction.

Where practical aid other than good advice is needed, it might well take the form of more nourishing food supplied regularly, and isolation for the invalid, with the disinfection of his clothes at a special consumptive laundry. Many people would like to see consumption made one of the maladies which is compulsorily notifiable to the sanitary authorities, when these preventive measures would probably necessarily follow; but until this is done, much may be accomplished by private effort to counteract the effects of ignorance amongst the class more especially liable to this illness, and to protect the relations of the sufferer from the consequences of their short-sighted economies. We often hear that a family is very consumptive, and that several of its members are threatened with the disease, when probably there was originally no organic reason why they should not all be strong and healthy. If one member of a family is attacked with consumption, there is no necessity for the others to get it too, provided that they can be taught to adopt sanitary conditions of living, and that they can be taught this without the intervention of the civic authority is very well illustrated by the success which Mlle. Chaptal has already achieved in this direction. Her results show what can be done by private endeavour, and surely there could be no better object to which to apply the funds of charity.

The day of "doles" is passing, we hope, but many philanthropically-minded people will still give 6d. or 1s. to anyone who asks for it, and probably a considerable sum is spent in a year on such "charity." If these doles could be collected and given to a specific object, such as this of aiding the thrifty poor to protect themselves from the ravages of this fell disease, how much good might not be done to the individual and to the public at large? How many might be saved from the threatened danger and kept in the ranks of the nation's workers? It is difficult to see what might not be the far-reaching results of such private efforts if only they were in the right direction, and, owing to the better sanitary knowledge and conditions now becoming general, consumption is said to be already slightly on the decrease.

### To Nurses.

We invite contributions from any of our readers, and shall be glad to pay for "Notes on News from the Nursing World," "Incidents in a Nurse's Life," or for articles describing nursing experiences at home or abroad dealing with any nursing question from an original point of view, according to length. The minimum payment is 5s. Contributions on topical subjects are specially welcome. Notices of appointments, letters, entertainments, presentations, and deaths are not paid for, but we are always glad to receive them. All rejected manuscripts are returned in due course, and all payments for manuscripts used are made as early as possible after the beginning of each quarter.

### The Two Matrons: An Australian Sketch.

"Matron of ours" was tall and beautiful to behold, she had clear kind eyes, a calm sweet face, a round firm chin, and a fine nose. She was always dressed in a neat close-fitting dark blue dress, with a snowy cap on her pretty grey hair, and snowy cuffs above her strong white hands. We all loved her, were her ardent admirers, her devoted slaves, yet how we trembled when marshalled for an admonition! how we grieved when detained for a reproof! how we rejoiced when praised for an effort to please or surprise her! We held her in great reverence and awe, for was she not full of justice and mercy? A guilty one once stood before her, stubborn, relentless, no reed shaken by the wind, but an old case convicted. She could not meet the cool stern gaze of the accuser; her eyes fell. She knew that the great woman stirred, came to her side; but she moved not. "Nurse!" thrilled the deep full voice, and before many moments had passed the hot tears fell on the girl's hard hands, and melted her harder heart. Suddenly she was at the matron's feet imploring forgiveness. She was forgiven, trusted, and proved worthy of the trust.

One of the matron's greatest attractions was her sense of humour, and her sympathy with our pleasure as well as in our pain. On one occasion one of the "big" doctors offered me a box at the theatre, which I sadly but emphatically refused with a shake of my head. He laughed, then suddenly left the room and returned with a broad grin on his face. "Matron is going to send you to the theatre to-night with a sister and two other 'good' nurses," he explained. She never refused any reasonable request, though she was terribly strict. Perhaps that is why the doctors admired her almost as much as we did, and called her a "splendid woman." The patients loved her too. When the men were dying they asked for her; when the women were sad, she would sit by their sides till the burden grew less. It was no drudgery to her to comfort and help the weak-hearted. And the children begged for her when they were happy and gay, or had new toys or games: never when they were in tears or pain; she never came to them then. Teddy, Sam, and Jean always kissed "matrum." How jealous we were of their hot little hands on her neck, of her smiles, and the sweet little things that she said to them! But we had our turn; it was when we said Good-bye. That blessing still remains in my heart, even unto this day—"Those things that you think good in me, go you and do likewise, for His sake."

• • • • • • •

Matron Number Two.

Fortune led me to a far country, to a place where there was a house called a "private hospital." The doctor had given permission to the patients' relations to send a nurse, and they sent me. While knocking at the front door, which was open, the first qualm overtook me—the whole place looked so dilapidated. Feeling inclined to fly, I encountered an old woman, who waved me upstairs smiling. I found afterwards that she always smiled. There were two rooms upstairs—one was mine, and in the other I found my patient, an unconscious child, by whom his mother sat holding his hot little hand. She started up and burst forth without preamble: "I am so glad you have come. These people never come near me; only once this morning the matron came with a cupful of weak beef-tea, and poured it down baby's throat. He has been sick ever since, and he cried so when she touched him; she was so rough, and her hands were dirty. He has not been washed, nor has his bed been made, and there is no clean linen."

"Must we stay here?" I inquired, attaching myself to them like a limpet to a rock.

"We are from the country; there is nowhere else, so the doctor sent us here."

"I have never nursed for Dr. H——. Do you trust him?" She said yes, so we determined to make the best of it. I went downstairs calling "Nurse," and the old woman appeared and led me into the kitchen. It was small and pokey; a boy was washing up in the sink, a very dirty baby was playing on the floor, and a nurse in very grubby uniform was standing in the dark by the stove stirring a kerosene-tin full of boiling clothes. "The baby upstairs," I stammered addressing the nurse. "Can you give me his doctor's instructions?"

"Yes," she said, coming forward, wiping her hands down her apron. I followed her on to the verandah, but she didn't say anything further.

"Well!" I inquired impatiently, facing her in the light. She was a big bony woman with a villainous expression, and a scar across her forehead. Her hair was tangled, and her cap, cuffs, and apron were crushed and soiled. "Well!" she said, "the same as usual. You are a nurse, you know." With this she stepped into the yard, splashed some cold water into a tub, put some soiled linen into it and rubbed it with soap. Then she dabbed it up and down for a minute, wrung it out with her hands, and went back to the kitchen to put it in the kerosene tin. I followed her asking for milk and some clean sheets. "Ask her for what you want," was all she vouchsafed, pointing to the smiling old woman, who was her sister. A nice and kind, though ignorant, old woman she was too—I discovered later, disgracefully overworked and put upon. She did all the housework and cooking of the hospital, and *also all the nursing.* You may not believe me, but it is a fact. The other only did the washing, wore the uniform, minded the dirty baby, and waited on the doctors. In the evening I descended the stairs once more to get some barley-water. In the kitchen stood a tiny woman in a black dress and a white apron. She wore no cap, and the top of her grey head was bald. Her whole demeanour denoted patience and long-suffering. I asked for the nurse.

"If you mean the matron," she replied quietly, but with great dignity, "she is out. So is the sister. I am in charge." I cannot describe the self-contained dignity, the defiant toss of the grey head, as the old lady said this, and I retired vanquished. It was altogether a mysterious hospital this, and grew more perplexing every day. There were no signs of any other patients, except an occasional cough or groan; there was no theatre, no bath-room, no sink—in fact, there was nothing. The dressings, medicines, and surgical appliances were kept in a packing-case on the verandah. One night I wanted a syringe in a hurry. The matron stood in the hall with the dirty baby on her arm, reading the evening paper. She threw me a "We haven't one," and went on reading.

"Are you sure?" I cried despairingly. It was late and the shops were shut.

"You can look in the box if you like!" The baby turned over her shoulder to watch me, his dirty round face laughing. I chuckled to him and he wriggled, trying to reach down with his chubby fists. This may have been very aggravating, but she need not have shaken him and made him cry. Hunting, with a red face, I found a syringe, which the matron said was broken. A squeeze at the end, a screw turned, and it was mended. I held it out to her triumphantly.

"It may be; I don't know," was all she said. "My sister always uses the things. I never do."

One day I heard a man gasping and groaning and crying out in agony as I passed his room. He sounded frightfully ill, and it was all I could do to prevent myself entering his room. Etiquette prevailed. I flew to the matron and told her.

"Bother!" she said. "There is the doctor, too, for the front room. I must go to him."

"But the patient!" I gasped.

"Oh, yes; tell my sister to go to him; she is in the kitchen." She was also dishing-up, but she put down the gravy-strainer with a sigh, and scurried off. That night the man died.

My baby grew so much worse that we gave up any hope of saving his life. The doctor had a consultation and told the mother this, so I stayed up all night with her, for she said that the woman in black, who was the night nurse, never came near them unless she was rung for, and that she was afraid of sleeping heavily. She slept in the child's room always. It was quite true: no one came near us all night, nor the next morning, though they knew how ill baby was.

When they called up that the breakfast was ready, I went downstairs, and found matron in a filthy wrapper. She never dressed till midday, and she did not even ask after baby, so I determined not to say anything, and to continue to sit up, which I did for five days and nights. By this time my head buzzed and my eyes ached, and I longed with an unholy longing for bath and bed. The doctor must have notice this, for he said abruptly:

"You had better go to bed, nurse."

"Who will look after baby?"

"I will tell the matron to."

I looked sullen—so did she when she came. I reported briefly, glanced at the mother sitting dejectedly by the child, met her agonised expression, went into my bedroom, looked at my dishevelled appearance in the glass, stretched my arms, groaned, and slunk back to baby. I could no more leave him than a cat its kitten. He was still unconscious, panting slightly; his mother lay sobbing on his pillow; the matron stood humming, crossly looking out of the window. I knelt down by the cot and felt his pulse. It was decidedly better; a watch told the same story. He breathed more naturally, turned over, smiled, and said "Mummie" softly. I gave him a drink and a hypodermic injection, then knelt down again at his feet. He went to sleep. I remember watching him with a glad feeling in my heart, but nothing more till it was dark. The doctor stood in the room; it was he who had touched my shoulder. There was a light, and I could see that he was smiling. The mother also was smiling.

"Look!" they said. "Baby is going to get well." I looked. The baby was smiling too.

### Cottage Hospitals and Some of their Difficulties.

BY A MATRON.

In working a cottage hospital by a trained nurse the two chief difficulties are a too large lay committee and a too small staff.

To take the first. Local feeling, perhaps, has something to do with the formation of a committee; the doctors are too busy to attend much, and the clergy sometimes have leisure, but their attendance is too erratic, and their holidays so long, that they are not ideal committee members. The ladies—generally elderly—have absolutely no knowledge of nursing. Often one has a strong will, and rules and over-rides the rest in committee. Consequently, when a hospital is opened, with five or six beds, the staff consists of one nurse-matron and one servant. No one knows what is needed, and no one is consulted. Nurses are to blame who take these posts, at salaries often no more than £25 a year. They know the position is unworkable, yet take it trusting that it may lead to better things. It is to be hoped that the time is not far distant when a hospital will not be opened with only one nurse, any more than with only one pair of sheets to each bed. It should be remembered that when one of the committee gets ill the conditions are reversed—there is one invalid and all the household to attend—yet one nurse is considered sufficient for five or six poor people. As a concession help is given in the form of a probationer, and this second difficulty is greater than the first.

A hospital is for the express purpose of tending sick people away from their homes, where they have no proper means of being nursed. They have the same doctors (in country districts) and the same treatment, but they pay—it may be only a half-crown a week, still they do pay to be nursed. Therefore it seems unfair that they should be handled by unskilled hands, and left in charge, as they must be sometimes, of a nurse who knows nothing of her work. The nurse-matron is responsible for the housekeeping, cooking, laundry, cleanliness of the wards, and the whole hospital, as well as the care of the patients; and part of her time must go to both parts of her duty. Then she is human, and needs rest and recreation, but a nurse working alone must leave some of these duties undone, though which it is difficult to answer. Servants are difficulties everywhere, but to go into the hospital seems to them a dreadful thing; perhaps in towns it may not be so bad, but in the far-off places, where the nursing work is just as constant, the cases just as bad, the people are still prejudiced. The cooks and girls still think the hospital a place of unknown horror.

Ladies in starting a cottage hospital should not dream of opening one until there are means sufficient to pay a decent salary for a fully trained matron, to give her an equally fully trained assistant nurse, and to pay a decent wage to a cook and one other servant; for unless good pay is given, inferior workers will be the only ones obtainable. Nursing, cooking, or laundry work is hard and monotonous; each is an honourable part of the whole. What would be the good of skilled nursing and no cooking or laundry?

Hospitals are improving year by year; vast sums are lavished on large institutions straining after perfection; these train the nurses who become the nurse-matrons of the small cottage hospitals. They are taught well, and strive to work their own hospitals to the credit of their nursing schools, and then they find the awful hardship of the insufficient staff. In individual cases one may lose heart, and not try to keep on; the other keeps on and ruins her health. If nurses could be sent from the large hospitals, in their third or fourth year, to work in cottage hospitals for three months, they would be benefited by learning self-reliance and many details which are lost in ward work, and the matrons of Cottage Hospitals would be helped by the fact of having a trained nurse with her, and being in touch with a medical school, would be able to hear of the most up-to-date improvements, and the methods of different doctors and surgeons. The advantages would be manifold. The cottage hospital in turn might take candidates too young for the training schools. Otherwise, probationers should not be taken in cottage hospitals any more than in expensive nursing homes. Lives are as valuable in high as in humble walks of life.

### In a Military Hospital.

BY A CIVIL SISTER.

It does not fall to everyone's lot to have the privilege of being shown over one of the military hospitals, so when I received a hearty invitation from some of the nursing sisters in one of the largest military stations to "come to tea and go round the wards," I was delighted.

Some years ago, when visiting the hospital, I was struck by the absolute bareness and want of decoration in the wards; clean, of course, they were, but withal uninviting. Now, the first thing that struck me was the evident signs of feminine handiwork and decoration. Masses of purple and white lilac, artistically arranged in green and white glass vases, greeted my eyes on entering the ward; they were not displayed on the ancient barrack-room wooden tables, but on modern, up-to-date green and white tiled tables, with brass-bound edges. The sister explained that these were a new importation, as were also many brilliantly polished copper articles.

The wards were all very fresh and airy, and on the top floor was the balcony for open-air cases, where the patients could lie out all day, and gaze at the most beautiful landscape in Surrey.

The floors were all polished with "ronuk" I was told. Poor sister gazed dolefully at a minute speck of fluff and sighed: "It is impossible to ever have the floor looking really nice; you know what Tommy Atkins is." Who would have him otherwise than the light-hearted, hard-working, frank fellow he is?

Sister laughed as she spoke, and turned to point out her youngest patient—a little fellow of about twelve, who seemed almost buried in the voluminous blue serge hospital suit he wore, in common with all the others.

We went into the scullery off the ward, where everything which could shine glowed like burnished gold or silver. "Do look at my pails," murmured a voice, and indeed no shield of ancient days was ever better kept than these humdrum everyday necessities. Downstairs we peeped into the ophthalmic ward, with blue glass windows, and then into the operating theatre. Strictly speaking there are two—an outer one, where septic cases are done and out-patient dressings applied, and a large inner one, kept as far as possible for clean cases, where everything is most up-to-date. Fortunately there was nothing going on at the time, so the corporal in charge courteously showed all there was to be seen, and pointed with pride to the new irrigating apparatus hanging over the table. I had a haunting recollection of instruments kept in velvet-lined cases, but I was introduced now to glass cupboards, where all the instruments that surgical hearts could desire were arranged in readiness for any emergency.

Before leaving I went into the little chapel, which is carefully tended by one of the sisters. At the east end, a carved wooden chair was placed, in memory of one of the nursing sisters who recently died at her post. Her funeral, I was told, was most striking and impressive, full military honours being given to her, who had indeed given all she possessed for others.

### Wants and Workers.

Nurse Howe, of 509 Holloway Road, N.—Will any kind friends help me with old uniforms? I am a fully trained nurse, and am married, but as my husband is unable to work at present, I must try and get nursing again.

### Everybody's Opinion.

[Correspondence on all subjects is invited, but we cannot in any way be responsible for the opinions expressed by our correspondents. No communication can be entertained if the name and address of the correspondent are not given as a guarantee of good faith, but not necessarily for publication. All correspondents should write on one side of the paper only.]

### SECTARIANISM IN LONDON HOSPITALS.

"Medicus" writes: As many rumours of sectarianism in hospitals have reached my ears from time to time, I should be glad to know whether it is really a fact that in most of the large London training schools a nurse professing the Catholic religion is, on that account, debarred from promotion to a sister's post. Perhaps some of your readers will kindly answer this question, either from their knowledge of these hospitals' constitutions, or from their own experience.

### AMERICA AND ENGLAND.

"Nurse A. S." writes: I should think an "American Nurse" did a wise thing when she returned to the States. Her letter is almost too contemptuous to take any notice of, save for the fact that she ends up with, "True it never happened in my case." As her statements were not written from personal experience, one would be glad to know through what channels her extraordinary knowledge was obtained.

"E. W." writes: I think the American nurse who wrote in your columns of August 25 was wise to return to America, and would do well to remain there, for she has evidently a wrong idea of nurses and nursing in England. I have been a nurse a good many years, and know several nursing homes, and although there are, and must be, some things at fault which the individual nurse cannot alter, yet I have never known a doctor to dress his housemaid in uniform, and send her to a "special" case for "special" fees. I am inclined to think that he would value his reputation too much to run such risks, for an untrained person could not be responsible for anyone who had undergone a serious operation. Perhaps the American nurse could suggest some course of action for us, as she thinks that nursing here is sadly in need of repair.

"A. K. R." writes: I am delighted to see that several of our English nurses have indignantly repudiated the slur that "An American Nurse" would cast upon our much-respected medical profession. Everyone is, of course aware, that there are a few black sheep in all callings of life; surely "An American Nurse" during her short experience in England must have met with them all, as far as the medical profession is concerned, or she could not make such absolutely false statements; or, can it be that she has tried to introduce too much American familiarity into her dealings with her superior officers and has met with a few kindly snubs from our self-respecting doctors? I think that few nurses who conscientiously carry out the instructions of the doctors for whom they work, and who recognise that their duty is to work under and with, not instead of them, would have cause to complain of the way they are treated by the medical profession. The training which "An American Nurse" has received may be excellent as far as it goes, but it is surely terribly lacking in one most essential point—namely, loyalty to her superior officers.

### THE "UNTRAINED HUMBUG."

"Seventeen Years' Attendant" writes: Taking The Hospital every week, I often read the abusive letters on the "Untrained Humbug" (as we are called). I am quite on the trained nurses' side. I know that there are untrained women who think of nothing but money and position, and, though untrained, go about describing themselves as trained nurses. They little know the mistake they make, for, as a rule, the trained nurse is much less bumptious and leaves other people to speak highly of her and her work. At least, this is my experience of several really clever nurses whom I have had the pleasure of helping. At the same time, we should not all be so lightly esteemed. I began my career as a maid to an old lady, always nursing her in common ailments, and when she became a confirmed invalid with paralysis I nursed her for four years, day and night, devoting myself to her up to her death. Though she could not speak for three years, I knew she was most grateful for all I did, and her very helplessness seemed to make me more anxious to do all I could for her. As I loved nursing and could not afford to be trained, I took up attendant's work, and I think untrained persons should stop at that, for however skilful we may be we cannot compete with our skilled sisters. We should remember, too, the years of hard work in hospital which they have to undergo before they can demand decent fees for their private work. I hope if this letter should be inserted that it will impress those who are working against their superior and more educated sisters, and also overcharging patients for untrained work. I am asked to wear uniform, but I never forget to let my patients know that I am only an attendant, untrained. Notwithstanding this fact, I am not afraid of not getting plenty of work in my own line.

### ESPRIT DE CORPS.

"L. S. R." writes: The subject of esprit de corps to which you referred the other day is one that has a wide and real significance not only for the nursing profession, and also for each individual member. It is an acknowledged and much to be deplored fact that there is lacking in a body of women the same spirit of loyalty which is to be found in a company of men. Time and space forbid me to go into the reasons for this, but nurses who care to analyse cause and effect will find in this ample material for half an hour's interesting and profitable thought. Esprit de corps! "The spirit of a body." All nurses who take their profession at all seriously will at once acknowledge that the spirit of a nursing body should include all that elevates and ennobles each member. The mere fact that all are servants in the very highest sense of the word should be sufficient to effectually settle the question of loyalty from one member to another and each to the whole. It matters nothing in what capacity we serve—whether as mental, fever, poor law, or general hospital nurses. All that matters is the spirit in which we, the servants of the sick, render our service. The sooner we recognise a common service and a common interest, with a spirit of loyalty permeating the whole, the sooner shall we evolve a higher and happier condition of things. Now, to get at the root of this particular evil, *i.e.*, disloyalty. As in everything else, it is the effect of a cause—easy to find, but harder to combat—character. It is surprising how much one strong character may do in this matter of loyalty. Who has not listened to a number of nurses discussing a fellow-nurse uncharitably, and heard the conversation entirely changed by some kindly remark from a woman whose character and personality forbade further comment? There are times, we know, when the only way to be loyal means silence, but in the majority of cases, talking to others about a particular black sheep will not whiten them (though occasionally a few kindly words said to them, from a real desire for their welfare, may help), so is it not wiser and much kinder to say nothing? Surely there is no breach of discipline in speaking a few kind words to a new probationer, or, if this be difficult to some temperaments, as it undoubtedly is, a kindly smile will be easier, and both are so acceptable! Nurses who have been through their training ought not to find it hard to give a kindly welcome to one joining a private nursing staff of which they are members. If it is, then there is lacking a very necessary element in a really successful nurse. A wise plan is to always keep our own first days green in Memory Lane. We shall then be in no danger of erring on the side of kindliness. This question of loyalty means so much to us nurses individually, because so much may be done by each one. The whole time of an institution, be it hospital or private nursing staff, can be entirely altered by a sprinkling of a few loyal women, for there is no leaven which works more quickly and more surely.

### Appointments.

[No charge is made for announcements under this head, and we are always glad to receive and publish appointments. The information, to insure accuracy, should be sent from the nurses themselves, and we cannot undertake to correct official announcements which may happen to be inaccurate. It is essential that in all cases the school of training should be given.]

Ashton-under-Lyne District Infirmary and Children's Hospital.—Miss Katharine F. Cooper has been appointed matron. She was trained at Northampton General Hospital, where she was afterwards sister. She has since been sister at the Metropolitan Hospital, London, theatre sister and assistant matron at Wolverhampton General Hospital.

Chapel-en-le-Frith Workhouse.—Miss Kezia Groundwell has been appointed charge nurse. She was trained at Bramley Union Workhouse.

Children's Sanatorium, Millfield, Rushington, near Worthing.—Miss S. A. Bodington has been appointed charge nurse. She was trained at the Victoria Hospital for Sick Children, Hull, and has since been charge nurse at the Orthopædic Hospital for Children, Redland, Bristol.

Fever Hospital, Smithy Bridge, Rochdale.—Miss Mary Grainger has been appointed nurse matron. She was trained at Manchester Royal Infirmary, and has since been matron at Ban, Morecambe.

Huddersfield Infirmary.—Miss Isabel M. Hall has been appointed night sister. She was trained at the New Hospital for Women, Euston Road, London, and St. Marylebone Infirmary. She has since been sister at St. Marylebone Infirmary.

Infectious Diseases Hospital, Ashton-in-Makerfield, Lancashire.—Miss C. E. Mulcahy has been appointed charge nurse. She was trained at St. George's Infirmary, London, S.W., where she was afterwards staff nurse. She has since been charge nurse at the Fullerton Accident Hospital, Rotherham, Yorkshire, and has done private nursing.

Perth Public Hospital, Western Australia.—Miss K. P. Ryan has been appointed matron. She was trained at Ballarat Hospital, where she was afterwards sister. She has since been matron of Maryborough Hospital.

Queen Victoria Jubilee Hospital, Melbourne.—Miss H. B. Samsing has been appointed matron. She was trained at the Melbourne Hospital, and has since done private nursing. She is a member of the Australian Army Nursing Service.

St. Nicholas's Home for Crippled Children.—Miss M. I. Gordon has been appointed assistant matron. She was trained at the County and City Royal Infirmary, Perth, N.B. She was sister with the Scottish National Red Cross Hospital at Kroonstad, South Africa; has done private nursing in Northallerton; been sister at the North Riding Infirmary, Middlesbrough; sister at the Birmingham and Midland Ear and Throat Hospital; night sister at St. Bartholomew's Hospital, Rochester; and sister at the Infirmary, Lewisham. She is a member of the Army Nursing Service Reserve.

### Notes and Queries.

**REGULATIONS.**

**The Editor is always willing to answer in this column, without any fee, all reasonable questions, as soon as possible.**

**But the following rules must be carefully observed.**

**1. Every communication must be accompanied by the name and address of the writer.**

**2. The question must always bear upon nursing, directly or indirectly.**

**If an answer is required by letter a fee of half-a-crown must be enclosed with the note containing the inquiry.**

*Registration.*

(256) Will nurses trained in small institutions, but who have been long in practice, have to be trained over again so as to be registered?

If a Registration Bill be passed we think that some provision will be made to place experienced nurses, wherever trained, on a just footing.

*Home for a Blind Person.*

(257) Can you tell me of a home in London where a blind gentlewoman could be received for a small weekly sum? Is there a club or guild for Roman Catholic nurses?—*Nurse.*

There is the Phœnix Home for Blind Women at 44 Alma Square, St. John's Wood, N.W., payment £20 a year, and the Home for Blind Gentlewomen at 69 Hanley Road, Finsbury Park, 13s. a week. For the Guild for Roman Catholic Nurses write to the Convent of the Visitation, Harrow-on-the-Hill.

*Midwifery.*

(258) How can I obtain the certificate of the Central Midwives Board?

Consult "How to Become a Certified Midwife," price 1s. 1d., from the Scientific Press, 28 Southampton Street, Strand, W.C.

*General Training.*

(259) How can I get twelve months' general training without unnecessary expense? I am slightly deaf; does that disqualify?—*L. A. B.*

You could go as a paying probationer to most hospitals for payment of about £1 per week. Your services might be accepted free in some institution, but for this you must advertise. Deafness is a great drawback to nursing.

*Army Nursing Service Reserve.*

(260) Please give me information as to the Up-Country Nursing Association, nursing in India and Egypt, sanitary inspectorship, British Nurses' Pension Fund.

The Up-Country Nursing Association is now merged in the Indian Nursing Association, and we should advise you to write direct to Lady Minto, the Viceregal Lodge, Simla, India. For nursing in Egypt apply to the Anglo-American or the Kasr-el-Ainy Hospitals, Cairo. For sanitary inspectorship write to Sanitary Inspectors' Examination Board, Adelaide Buildings, London Bridge, E.C. Do you mean the fund attached to the Royal British Nurses' Association? If so, address the Secretary at 10 Orchard Street, Oxford Street, W. If the Royal National Pension Fund for Nurses, write to the Secretary, 28 Finsbury Pavement, E.C.

*Norland Institute.*

(261) *Bedelia* sends no name. See our rules.

*Midwives Defence Union.*

(262) I saw the institution of such a union advocated in your paper. Has one been formed?—*A Certified Midwife.*

No union has yet been formed, and probably so great difficulty will be found in securing the co-operation of the midwives themselves, that unless some recognised institution takes the matter up such an organisation will never be established.

*Unregistered Midwives.*

(263) Does the Central Midwives Board allow unregistered midwives to practise? Is it possible for a holder of the L.O.S. certificate to still register without passing the C.M.B. examination?—*Bristol.*

No woman may practise as a midwife without the C.M.B. certificate. You cannot register except through the C.M.B.

**Handbooks for Nurses.**

| | Post Free. |
|---|---|
| "A Handbook for Nurses." (Dr. J. K. Watson) ... | 5s. 4d. |
| "Nurses' Pronouncing Dictionary of Medical Terms" | 2s. 0d. |
| "Art of Massage." (Creighton Hale) ... ... ... | 6s. 0d. |
| "Surgical Bandaging and Dressings." (Johnson Smith.) ... ... ... ... ... ... | 2s. 0d. |
| "Hints on Tropical Fevers." (Sister Pollard.) ... | 1s. 8d. |

Of all booksellers or of The Scientific Press, Limited, 28 & 29 Southampton Street, Strand, London, W.C.

### For Reading to the Sick.

"THY KINGDOM COME."

Ah! the dream!
All fair, could it but last in waking hours!
Could men but hear the angels' song anew,
And learn to sing it, making "Peace on earth!"

Peace beginning to be
Deep as the sleep of the sea,
When the stars their faces glass
In its blue tranquillity:
Hearts of men upon earth,
Never once still from their birth,
To rest, as the wild waters rest,
With the colours of heaven on their breast!

Love, which is sunlight of peace
Age by age to increase,
Till Angers and Hatreds are dead,
And Sorrow and Death shall cease:
"Peace on earth and goodwill!"
Souls that are gentle and still
Hear the first music of this
Far-off, infinite bliss!

*Sir Edwin Arnold.*

This is Christianity, a spiritual society, not because it has no worldly concerns, but because all its members, as such, are born of the Spirit, kept alive, animated and governed by the Spirit of God. It is constantly called by our Lord the kingdom of God, or heaven, because all its ministry and service, all that is done in it, is done in obedience and subjection to that Spirit, by which angels live, and are governed in heaven. Hence our blessed Lord taught His disciples to pray that this kingdom might come, that so God's will might "be done on earth, as it is in heaven"; which could not be, but by that same Spirit, by which it is done in heaven.—*William Law.*

If the outward were the measure of the Church of Christ, we might, as we have seen, well despair. But side by side with us, when we fondly think, like Elijah or Elisha's servant, that we stand alone, are countless multitudes whom we know not, angels whom we have no power to discern, children of God whom we have not learnt to recognise. We have come to the kingdom of God, peopled with armies of angels and men working for us and with us, because they are working for Him. And though we cannot grasp the fullness of the truth, and free ourselves from the fetters of sense, yet we can, in the Light of the Incarnation, feel the fact of this unseen fellowship; we can feel that heaven is being re-opened by Christ.

Beyond all we may surely reckon upon the personal coming of our dearest friends to meet us, when we are loosening from the house of our mortality, and going forth to join them. For we must believe it to be a law of our Father's House, that whoever are most akin to us, most in sympathy with us, and most inwound with our affections, will come to meet us in the air, and bear us along love's way, to our own home.

The nearest of kin to our inmost souls may be persons whom we have never yet seen. Not till death did Lazarus see his angel-friends, who carried him to the society of his eternal kindred. Exquisite rest! Fullness of joy in the satisfaction of Love!—*Dr. J. Pulsford.*

